# Loot

**Published:** 1872-08

**Authors:** 


### Loot.

***A Nervous Atmosphere.***

A theory has lately been propounded by Dr. B. W. Richardson, of London, which is in some sense a return to the old view of a nervous fluid, and in some sense also is an extension to the nervous system of the physical idea of communications of motion by molecular disturbance. He supposes that the blood, as it circulates through the vessels, yields a diffusible vapor or atmosphere which charges the nervous system, surrounding the molecules of nervous matter and pervading the whole nervous organism. He attempts to formulate the physical qualities of this vapor; it is probably an organic vapor containing carbon, hydrogen, and nitrogen; it is insoluble in blood, it is condensible by cold, diffusible by heat; it possesses conducting power, and as a physical substance is susceptible of variations of pressure; it connects the nervous system in all its parts together; it is the medium of communication during life between the outer and the inner existence; by the organs of the senses the impressions and motions derived from the outer world are vibrated into or through the nervous atmosphere to the brain; in the living and healthy animal the nervous ether, if we may so designate it, is in correct tension, in the feeble it is diminished, in the dead it is absent or inactive; in the waking times of the living it is most active; it may be used up faster than it is produced during exercise; it is renewed during sleep.

On the supposition of the existence of a nervous ether or atmosphere, the author accounts for various phenomena. It is assumed that vapors of chloroform or alcohol, for example, taken into the blood and carried to the nervous system, become diffused through the nervous atmosphere. "The foreign vapor that has been introduced benumbs: in other words, it interferes with the physical conduction of impressions through what should be the cloudless atmosphere between the outer and inner existence." The rapid action of certain poisons, and the diffusion of the products of decomposition generated within the body itself, in disease, and the sudden collapse of nervous action which is often seen, are similarly accounted for by the author of the theory.

***The Modern Idea of Disease.***

In an address before the Clinical Society of London, Sir William Gull remarks as follows on the modern idea of the nature of disease:

Respecting the object we work for,—this living organism of ours,—one great advance has of late been made. We are acquiring a physiological notion of disease. Disease is no entity; it is but a modification of health,—a perverted physiological process; and this must at all times be insisted upon. Were it not that we fear death and dislike pain, we should not look upon disease as anything abnormal in the life-process, but to be as part and parcel of it. Few would now venture on a definition of disease; for in reality it is but the course of nature in a living thing which is not health. In health, the balance of function is even; incline it to either side, and there is disease. That being so, just as the life-process constitutes an individual and puts him apart from his fellows, so must any alteration in it be individual, and not general. But to the ignorant, disease is an entity,—an evil spirit which attacks us and seizes us. Hence arises the word "seizure," which, though in a somewhat different way, we still use; but with a protest. To the charlatan, disease is a set of symptoms to be attacked by a variety of drugs,—a drug for each symptom. To us, disease is a life-process of a perverted kind.

Many states are not now called diseases which used to be, and there are still some to be expunged. Some people are always ailing. Some have feeble stability, and to them it is as natural to be ill as it is to others to be well; but this is not disease. So, too, aged persons get ill; but this is not disease,—in reality it is natural change simulating disease, and when we try to cure such, we use all the farrago of the chemist's shop to prevent the sun setting. So syphilis at last ceases in the system to be syphilis, and becomes an early decay.

It is curious to consider the various morbific agents at work within our bodies, the lines in which they work, and their seats of action. These as yet have been but little studied, and deserve attention. Thus, it is very doubtful if scarlatina begins in the blood, as we should all be apt to say, rather than in any other tissue or fluid. Let it be our object to find out where all these begin within the body, and how they enter the body.

In future, I hope, comparative pathology, which is just beginning to be studied, will teach us much; for in our bodies we men have many organs which are of little or no use to us, and are only relics of a former state of being. What, for instance, is the comparative anatomy of the tonsils? Were I to make a man, I do not think I would put tonsils in him. Yet these, and such like organs, in accordance with the general law, are more prone to disease than are the others which are of real use in the system. I remember the case of a man who had a permanent vitelline duct. He had been out on a cold day, and the motion of the intestines twisted them in a mass round his persistent duct, and he died. I made a preparation of the duct, and wrote under it, "Cui vitam atque mortem dedit diverticulum." Every part of the body is alive, and has its own individual life and pathology, whether it be immediately required or not; only, if not required, it is more prone to disease than if it were. I could, for instance, suppose a fœtus of four months going to the doctor and saying, "I am going all wrong; my Wolffian bodies are disappearing, and kidneys are coming in their stead." Yet that is as much a condition of disease as some of those conditions of which I speak.

### *Treatment of Carbuncles.*

Carbuncles, according to the *Georgia Medical Companion*, are most safely, humanely, and more regularly treated as follows:

Introduce the canula of a hypodermic syringe into the centre of the tumor, draw out the piston, and with it will come pus, if any be present. The syringe is to be removed from the canula and emptied, the canula left in, and the syringe replaced to the canula again, and the piston withdrawn as before, as long as pus follows. When all the pus is out, withdraw the canula and apply on the tumor, externally, with a brush, the following:

| | | |
|---|---|---|
| R. | Collodion, - - - - - - - | dr. i. |
| | Castor Oil, - - - - - - - | gtts. xx. |
| | Carbolic Acid, - - - - - - - | grs. v. |
| | Tannin, - - - - - - - - | scr. i. |

Mix. Several applications are to be made, one after the other, so that a good outer covering is obtained at once. If the patient is weak, give him tr. iron, 20 drops, every four hours; also,

| | | |
|---|---|---|
| R. | Fl. Ext. Peruvian Bark, - - - - | gtts. xx. |
| | Spts. Ammon. Arom , - - - - | grs. xx. |
| | Infusion Gentian, - - - - - | oz. i. |

Mix. Repeat as often as deemed advisable.

***Feeding by the Rectum.***

The feeding of patients by nutritive enemata in cases of stricture of the œsophagus or pylorus, or whenever the upper part of the digestive tube must be relieved of its functions, has hitherto been anything but a satisfactory proceeding. The ordinary fluid food which is generally used for that purpose is either not retained long enough in the rectum, or cannot be digested there for want of a digestive ferment. Even the proposal of Meissner, to use an artificially prepared meat-peptone, has not always been found practicable, and the long time necessary for its preparation makes it quite unsuitable for daily practice. A decided step in advance has recently been made by Dr. W. O. Leube of Erlangen (*Deutsches Archiv fur Klin. Medic.*, vol. x.) Starting from the idea that it would be best to let the digestive changes which must necessarily precede absorption go on in the rectum itself, with its equable temperature, he devised a mixture of food and digestive ferment which, he found, is easily retained in the rectum from twelve to thirty-six hours. The digestive ferment is the fresh pancreas of the ox or pig, which, finely minced, he mixed with scraped meat, rubbing them well together with a little warm water, so that the mass may be easily injected. The most suitable proportion is one part of pancreas to three of meat. Fat may be added, but its quantity ought not to exceed one-sixth of that of the meat. Before this food is injected, the rectum ought to be washed out with water. Dr. Leube mentions that the first enemata sometimes apparently remain undigested, but that this must not prevent their being continued. Generally the fæces resulting when this food has been retained sufficiently long, have the character of ordinary fæcal matter. By a series of experiments, Dr. Leube has proved that by this method of feeding *per rectum* a considerable quantity of nitrogen is taken up into the system. In a dog, which for several days had been deprived of nitrogenous food, and whose system, therefore, was in a state of nitrogen-hunger, an increase in the nitrogen-elimination by the kidneys took place when these nutritive enemata were given; and, on the other hand, in several experiments on a dog, and likewise on a healthy young man whose system was in a state of satiation with regard to nitrogen, the quantity of nitrogen excreted through the kindeys was not materially diminished when most of the nitrogenous food was introduced by the rectum instead of the stomach. A chemical examination of the fæces remaining when the food had been retained long enough, showed that almost the entire quantity of nitrogen contained in the food had disappeared. The same was found with regard to fat; and in a dog that was killed on the second day of the experiment, the epithelial cells of the mucous membrane of the colon were found filled with fat globules. Dr. Leube also relates three cases of patients in whom this method of feeding had been used, and has completely answered the expectations which had been formed from his experiments. Of particular interest is the last case, in which, in consequence of tincture of iodine having been accidentally swallowed, no food whatever could be taken by the stomach, and the feeding by the rectum had to be continued for more than four weeks. In all three cases the general condition of the patients was much improved, although the nature of the cases precluded any but temporary benefit, two of the patients suffering from carcinoma.—*British Medical Journal.*

***Cold Alcoholic Test for Albumen.***

Dr. C. R. Drysdale reports from the Metropolitan Free Hospital, that he has tried the cold alcohol test for albumen, recommended by Dr. Betz (*Memorabilien*, 1872,) and that it has proved trustworthy in the cases tried by him. Dr. Betz remarks that boiling the urine is not a sufficient plan when examinations are made in private practice, because albumen is not always thrown down by boiling. Also nitric acid is not certain in all cases. He mentions that a trustworthy and very easily obtained reagent is ordinary spirit as bought in shops. A portion of the urine is poured into a glass, and over it about an equal quantity of spirit, without allowing the two liquids to mingle. When albumen is present, the alcohol has a milky haze at the junction, and occasionally there are small nipples of albumen seen in the alcohol when the urine is very full of it. This process is so simple that it can always be made use of. According to Dr. Betz, this test will frequently show albumen when we are not inclined to think it exists, on account of the absence of œdema, heaviness of the body (which is seen in children,) foaming of the urine on micturition, scarlatina or pneumonia. Dr. Drysdale has found the reaction in four cases of chronic albuminuria now under his care at the hospital.—*Medical Press and Circular.*

***Electrolysis in Ovarian Tumors.***

Dr. F. Fieber reports (*Wien Med. Presse*) the following: "A seamstress, aged 32 years, had a tumor of the size of a man's head, slightly painful on pressure, hard, nodulated, extending from half an inch below the umbilicus down to within an inch above the symphysis; its breadth was six inches. Diagnosis: ovarian cyst of the right side.

"June 6th, 1870, a large gold needle with a copper pole, connecting with Daniel's battery of twenty elements, was inserted one and a half inches deep into the swelling, one and a half inches below and to the right of the umbilicus, the chain was then closed to the left of the umbilicus. While the needle was inserted, the patient experienced a sensation of severe burning, which, however, soon disappeared; after seven minutes the needle was withdrawn; reaction slight.

"Up to November 30th, electrolysis was repeated eleven times, subsequent inflammatory symptoms but rarely requiring poultices and small doses of morphine. The needle was inserted into different places. Till the end of November, the swelling diminished but little; afterward it decreased rapidly. March 10th, 1871, the cyst was reduced to the size of a hen's egg."

Dr. F. urgently recommends in every case the employment of electrolysis before undertaking ovariotomy.—*Medical Archives.*

***Michel's Process for Removing External Tumors.***—By Dr. William A. Bell.

A little pamphlet gives an interesting account of the mode of operation for the removal of tumors practiced by a French charlatan, for a knowledge of which Dr. Bell paid no less a sum than 25,000 francs, and which, having now obtained complete information, he has very properly and liberally made public. The preparation used in all cases where the tumor can with safety be reached externally is made in the following way: Asbestos, as soft and free from grit as possible, is reduced by rubbing between the hands to the finest possible fleecy powder; it is then mixed thoroughly with three times its own weight of strong sulphuric acid ($S O^3 H O$). A mass is thus formed which may be easily worked with a silver or gold spatula into any size or shape, corresponding to the tumor to be destroyed. Any malignant growth of the breast which is detached and solitary, with the subaxillary glands unaffected, is suitable for treatment, whether open or not makes no difference. In the application of the caustic the adjoining healthy parts of the skin are carefully protected by applying a zone of collodion and pads of linen, and the patient is so placed that the surface of the tumor is perfectly level. The saturated acid-asbestos is then laid on the surface to the thickness of half an inch for a tumor the size of a hen's egg. Rapid destruction of the tissues follows, with, after the first half-hour or so, but little pain. An oozing of clear watery fluid appears, which must be carefully sopped up. After twelve or fourteen hours' action, the first application is to be removed, and a new portion of smaller size adapted to the sore. After this has been applied for twelve hours, the operation is complete, and the healing of the deep excavation alone requires to be attended to, for the details of which we must refer our readers to the pamphlet. Dr. Bell does not pretend to say that this mode of operation will effect a permanent cure of cancerous cases, but he thinks that the plan presents various and considerable advantages over extirpation by the knife, as in producing much less shock to the system, in removing the tumor alone with but little of the surrounding breast, and in postponing, in malignant cases, for a longer period the recurrence of the disease.—*Practitioner.*

***Ears versus Brains.***

Prof. Lacock says that the form of the ear depends upon two fundamental elements, namely: First, the cartilage, with its muscles; secondly, the helix and lobule. In man, the cartilage of the perfect ear is comprised within an ellipse or ellipsoid proportionate to the head, and to this is attached a geometrically formed helix and a pendent ellipsoid lobule. In proportion as these parts are defective, or as the ear is monstrous, triangular, square, or of an irregular form, it indicates a tendency to cerebral degeneration or defect. Monstrous ears, with defective helix or lobules, are very common in idiots. Men of high intellectual attainments, great capacity for mental labor, and great force of character, have a full perfectly ovoid ear, and the helix well developed, the lobule plump, pendent and unattached to the cheek at its anterior margin. These characteristics are seen in all the portraits of great men which Lavater gives, and are easily observed in living celebrities. In a perfect ear the ovoid lobule hangs from the cartilage with a rounded lower margin, which at its inner border is not confluent with the face. If this inner margin be adherent to the cheek, and at the same time the lobule be only a segment of an ellipse, there is more or less tendency to imperfect cerebral action.—*Lond. Med. Times and Gazette.*—*Detroit Review.*

---

***Prize Essays.***

The Committee of the American Medical Editors' Association, appointed on a proper subject for prize essays, have recommended the following for the prize to be awarded in May, 1873:

"The Pathology and Treatment of Diseases of the Ovaries."

And for the prize to be awarded in May, 1874, the following:

"At what stages of Pulmonary Tuberculosis is a change of climate desirable; what are the principles which should govern us in chosing the kind of change to be made; and the best localities in North America to send patients of this class?"

The prize offered is $100, and competition is open to all medical editors.—*Boston M. and S. Journal.*

